# Privacy-protecting, reliable response data discovery using COVID-19 patient observations

**DOI:** 10.1093/jamia/ocab054

**Published:** 2021-05-29

**Authors:** Jihoon Kim, Larissa Neumann, Paulina Paul, Michele E Day, Michael Aratow, Douglas S Bell, Jason N Doctor, Ludwig C Hinske, Xiaoqian Jiang, Katherine K Kim, Michael E Matheny, Daniella Meeker, Mark J Pletcher, Lisa M Schilling, Spencer SooHoo, Hua Xu, Kai Zheng, Lucila Ohno-Machado, for the R2D2 Consortium

**Affiliations:** 1 UC San Diego Health Department of Biomedical Informatics, University of California San Diego, La Jolla, California, USA; 2 Institute for Medical Information Processing, Biometry, and Epidemiology, Ludwig Maximilian University of Munich, Munich, Germany; 3 LMU Klinikum, Department of Anesthesiology, Ludwig Maximilian University of Munich, Munich, Germany; 4 San Mateo Medical Center, San Mateo, California, USA; 5 Biomedical Informatics Program, UCLA Clinical and Translational Science Institute (CTSI), Los Angeles, California, USA; 6 USC Schaeffer Center for Health Policy and Economics, Price School of Policy, University of Southern California, Los Angeles, California, USA; 7 School of Biomedical Informatics, The University of Texas Health Science Center at Houston, Houston, Texas, USA; 8 Betty Irene Moore School of Nursing, University of California Davis Medical Center, Sacramento, California, USA; 9 Health Informatics Division, Department of Public Health Sciences, School of Medicine, UC Davis Health, Sacramento, California, USA; 10 GRECC Tennessee Valley Healthcare System, Nashville, Tennessee, USA; 11 Department of Biomedical Informatics, Vanderbilt University Medical Center, Nashville, Tennessee, USA; 12 Department of Preventive Medicine, Keck School of Medicine of USC, Los Angeles, California, USA; 13 Department of Epidemiology and Biostatistics, University of California, San Francisco, San Francisco, California, USA; 14 Data Science and Patient Value Program, University of Colorado Anschutz Medical Campus, Aurora, Colorado, USA; 15 Division of Informatics, Department of Biomedical Sciences, Cedars Sinai Medical Center, Los Angeles, California, USA; 16 Department of Informatics, Donald Bren School of Information and Computer Sciences, University of California, Irvine, Irvine, California, USA; 17 Veteran Affairs San Diego Healthcare System, San Diego, California, USA

**Keywords:** COVID-19, observational study, common data elements, electronic health record, regression analysis

## Abstract

**Objective:**

To utilize, in an individual and institutional privacy-preserving manner, electronic health record (EHR) data from 202 hospitals by analyzing answers to COVID-19-related questions and posting these answers online.

**Materials and Methods:**

We developed a distributed, federated network of 12 health systems that harmonized their EHRs and submitted aggregate answers to consortia questions posted at https://www.covid19questions.org. Our consortium developed processes and implemented distributed algorithms to produce answers to a variety of questions. We were able to generate counts, descriptive statistics, and build a multivariate, iterative regression model without centralizing individual-level data.

**Results:**

Our public website contains answers to various clinical questions, a web form for users to ask questions in natural language, and a list of items that are currently pending responses. The results show, for example, that patients who were taking angiotensin-converting enzyme inhibitors and angiotensin II receptor blockers, within the year before admission, had lower unadjusted in-hospital mortality rates. We also showed that, when adjusted for, age, sex, and ethnicity were not significantly associated with mortality. We demonstrated that it is possible to answer questions about COVID-19 using EHR data from systems that have different policies and must follow various regulations, without moving data out of their health systems.

**Discussion and Conclusions:**

We present an alternative or a complement to centralized COVID-19 registries of EHR data. We can use multivariate distributed logistic regression on observations recorded in the process of care to generate results without transferring individual-level data outside the health systems.

## INTRODUCTION

The COVID-19 pandemic represents a watershed event in public health and has highlighted numerous opportunities and needs in clinical and public health informatics infrastructure.^1–3^ One of the key challenges is the rapid response of analyses and interpretation of observational data to inform clinical decision-making and patient expectations, understanding, and perceptions.^4–8^

Several initiatives are building COVID-19 registries or consortia to analyze electronic health record (EHR) data.^7^ The expectation is that these resources will provide researchers and clinicians access to a rich source of observational data to understand the clinical progression of COVID-19, to estimate the impact of therapies, and to make predictions regarding outcomes. Registries may contain limited data for patients diagnosed with COVID-19: the barriers to having more data are based on both privacy concerns and on what elements have been deemed valuable by health professionals and researchers at a particular point in time. The problems with a new and evolving disease like COVID-19 is that we do not know what data or information will be most valuable. For example, in the pandemic’s early stages, the dermatological and hematological findings were not evident, and those data were not included in registries or reports.^9^ Interest in specific laboratory markers (eg, D-dimer, troponin) for these disturbances and additional medications (eg, antihypertensive drugs) or phenotypes (eg, diabetes, blood type) has increased over time.^10–12^ Additionally, it is challenging for researchers and clinicians to understand the structure and quality of the data in data repositories, and to formulate queries to consult the data in their institution and in others.

Thus, the utilization of EHRs to characterize COVID-19 disease progression and outcomes is challenging. However, EHR data may be useful when a randomized clinical trial cannot be conducted. Observational data may also help determine if results from a randomized clinical trial replicate after relaxing eligibility criteria for real-world applications. While the scientific community has raised concerns about the reproducibility of findings, data provenance, and proper utilization of observational data, resulting in some COVID-19 articles being retracted,^13^ there remains a clear need to responsibly, ethically, and transparently analyze observational data to provide hypothesis generation and guidance in the pursuit of evidence-based healthcare.

In this study, we focus on using novel decentralized data governance and methods to analyze EHR-derived data.

## MATERIALS AND METHODS

Researchers’ questions posed in natural language are answered by distributed data maintained in 12 health systems, covering 202 hospitals located in all US states and two territories and one international academic medical center (Table 1). This collaboration provides the capability for comparisons with historical data from over 45 million patients and uses a dynamic approach to account for an evolving awareness of the most impactful COVID-19 questions to answer and hypotheses to explore. All sites have transformed or are actively transforming data into the Observational Medical Outcomes Partnership Common Data Model (OMOP CDM), but some of them only use data from COVID-19 registry patients (ie, do not transform the full EHR-based data warehouse), and others only have the items required by the query in OMOP. The ability to build and evaluate multivariate models across a large number of health systems and integrate results from registries differentiates our approach from most federated clinical data research network approaches.

**Table 1.** Participating sites: Cedars Sinai Medical Center (CSMC), University of Colorado Anschutz Medical Campus (CU-AMC), Ludwig Maximilian University of Munich (LMU), San Mateo Medical Center (SMMC), University of California (UC) Davis (UCD), Irvine (UCI), San Diego (UCSD), San Francisco (UCSF), University of Southern California (USC), University of Texas Health Science Center at Houston and Memorial Hermann Health System (UTH), Veterans Affairs Medical Center (VAMC)

**Institution** ^b^	Hospitals	Beds	Discharges per year	EHR system	Data source
CSMC	2	1019	61 386	Epic	EHR
CU-AMC	12	1829	106 325	Epic	EHR
LMU^a^	12	1964	78 673	SAP/i.s.h.medQCare IMESO	COVID-19 Registry
SMMC	1	62	1951	Harris Software(Pulsecheck)Cerner (Soarian)eClinicalworks	EHR
UCD	1	620	32 248	Epic	EHR
UCI	1	417	21 656	Epic	EHR
UCLA	2	786	47 491	Epic	EHR
UCSD	3	808	29 895	Epic	EHR
UCSF	3	796	48 120	Epic	EHR
USC	2	1511	23 454	Cerner	EHR
UTH	17	4164	233 890	Cerner	COVID-19 Registry
VAMC	146	13 000	676 402	ViSTa/CPRS	EHR
Total	202	26 976	1 361 491		

a Available data on hospital characteristics from 2018.

b Two additional sites joined the consortium and will begin answering queries in 2021.

The development of our Q&A system involved the inclusion of new concept codes in local repositories, agreement on concept definitions (eg, what constitutes a *COVID-19 hospitalization*, what codes should be included in the definition of *History of Coronary Heart Disease*, and how to map laboratory test records into LOINC, for which we developed a mapping tool).^14^ Instead of a singular control of a coordinating center, the R2D2 consortium allows participating institutions to “own” the development and testing of queries across various sites, which promotes a balanced division of workload and increases the ability of individual sites to develop generalizable queries and manage responses with help from the whole consortium. The translation of questions into code relies on members of the Reliable Response Data Discovery for COVID-19 (R2D2) Consortium. The analyses performed on data transformed into the OMOP CDM from relevant patient cohorts do not require data transfer outside the participating institutions and reduce the risk of individual or institutional privacy breaches. After a partially automated quality control process, which is carefully reviewed by multiple consortium members, only the results of calculations (eg, counts, statistics, coefficients, variance–covariance matrices) are released from the healthcare institutions; no individual patient-level data are shared.^15^

### Workflow

**Figure 1.**
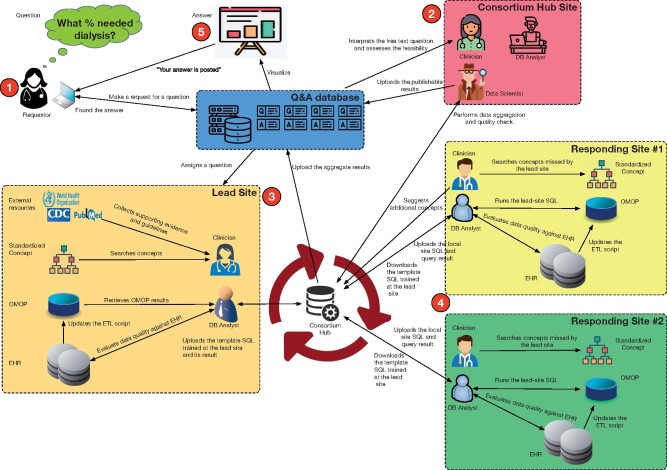
**What happens behind the scenes: from questions to answers**. The workflow of the question-answer system is shown in 5 steps. Step 1. Users access a public web portal and post a new question if they cannot find a posted answer. Step 2. The questions get triaged to a Consortium Hub clinical informatician who determines their general interest and assigns the edited version of the question to a Lead Site. Step 3. At the Lead Site, the clinical informatician and the database analyst work together to create concept sets, design a query, and check local results. Step 4. The Responding Site runs the released structured query language (SQL) code and uploads its results to the Consortium Hub. During this step, the clinical informatician and the Responding Site data analyst adjust the concept set, inclusion logic, and database query code in SQL for local implementation; obtain and quality control the site-level results; and submit results to the Consortium Hub. Step 5. The Consortium Hub aggregates the site-level results, generates the visualizations, and posts the answer on the web portal.

**Figure 2.**
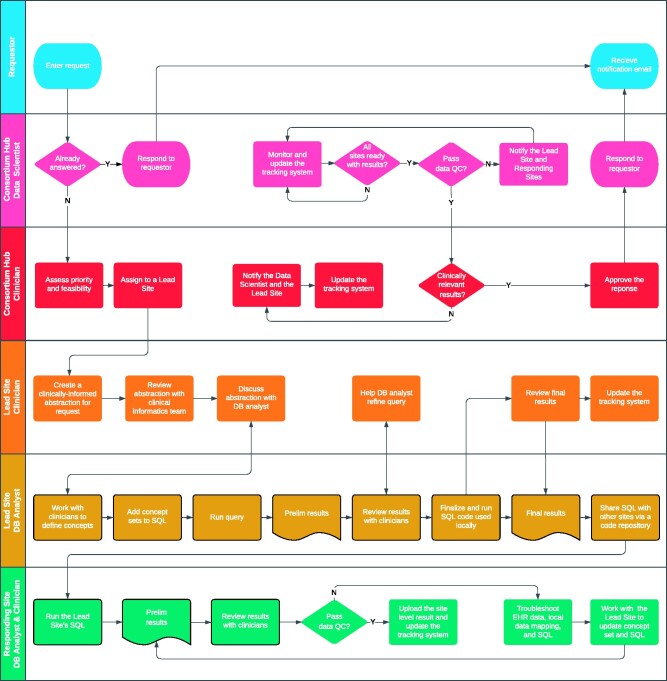
**Swimlane diagram**. A Q&A process flow starts from a user entering a request and ends with the user receiving e-mail notification about a response. At the Consortium Hub, the data scientist is responsible for aggregating site-level results and for data quality checks. The clinician at the Consortium Hub is responsible for feasibility assessment of the question, triaging to a Lead Site, and for the approval of the aggregate answer. At the Lead Site, the clinician reviews the assigned question text and works with the database analyst to translate the question into SQL and ensure the results are clinically relevant. The database analyst at the Lead Site writes the SQL code, runs it, verifies the results, and releases the code to the Consortium Hub. At the Responding Site, the database analyst runs the Lead Site’s SQL code, reviews the results together with local clinicians, and uploads the site-level results to the Consortium Hub through an iterative process of ETL update, local data mapping, and concept set development led by the Lead Site.

Figure 1 shows our general workflow, including human interpretation and clarification of questions and human quality control of answers, using graphs and related visualizations as much as possible. The responsibility of the Lead Site—to create a template query for all responding sites to use for rapid response—rotates among institutions (ie, health systems). A more detailed workflow is illustrated in Figure 2 using a swim-lane format with an emphasis on roles. The Q&A process starts when a user creates a request through the public website, https://covid19questions.org. Next, the data scientist at the Consortium Hub verifies whether this question had been answered before and passes it on to the clinician at the Consortium Hub to assess the feasibility (ie, if the received question is answerable from the local data mart), who then assigns it to one of the 12 institutions as a Lead Site. Throughout the whole process, the tracking system is used to report an issue to assignees, respond to the issue, update the code and results, and prompt to rerun the updated structured query language (SQL). Next, another clinician at the Lead Site works with the local database analyst to review and develop a concept set. This is an iterative process within the Lead Site: to develop a concept set, create SQL, generate results, and evaluate the results against the EHR records, including chart reviews. The outputs of the Lead Site-level process are a template query (.sql format) and a template output (.csv format), which are uploaded to the shared code repository.

**Figure 3.**
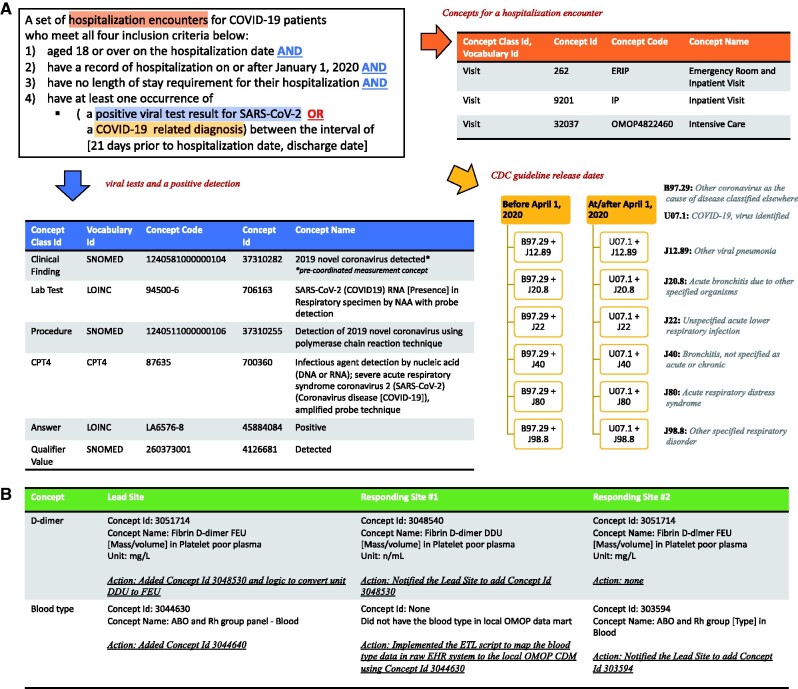
**Cohort definition and concept set development**. Defining a cohort of patients that is frequently used to answer questions helps us reuse code. In this example, defining the cohort of patients hospitalized with COVID-19 involves use of SARS-CoV-2 test results or diagnosis codes (A). In (B), we illustrate how a laboratory test is defined differently at two sites and how blood type had yet to be harmonized into OMOP at one site.

Once 11 Responding Sites get notification e-mails about the template query and format for the results, their database analysts will run the template SQL to get preliminary results and review these against their EHR data with clinicians. This part of the process is where the Responding Site most frequently runs into errors and challenges and requires troubleshooting. For example, when missing concepts, like D-dimer or blood type (illustrated in Figure 3), are discovered, the database analyst at the Responding Site creates an issue in the tracking system and resolves this with the database analyst and the clinician at the Leading Site. Since there are 11 Responding Sites, this means the Lead Site coordinates the concept set and SQL development through one-on-one sessions between the Lead Site and Responding Site. Through this iterative process among 12 sites, the concept set and SQL are continuously updated, improving their sensitivity and specificity to identify the right patients and hospitalization encounter records. This involves rewriting and updating existing extract-transform-load (ETL) scripts to map source EHR data to target the common data model (CDM, which in our case is the Observational Medical Outcomes Partnership, OMOP).^16^ The institutions with the same EHR system or database management system share common experience and knowledge to help each other develop ETL scripts together and evaluate the OMOP query results against EHRs.

**Figure 4.**
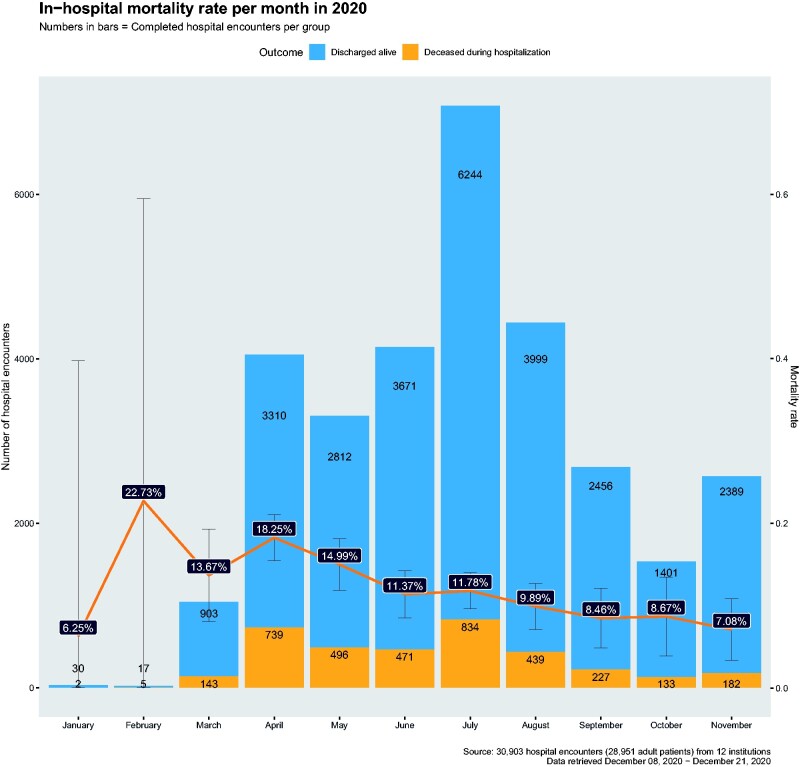
**An example of a COVID-19 question: monthly mortality**. The in-hospital mortality rate per month (red line) is shown as a percentage, with its 95% confidence interval between January and November in 2020. The observed counts for the deceased during hospitalization (orange) and the discharged alive (blue) are shown in bar plots. The unit of analysis is the hospital encounter.

When all Responding Sites have uploaded their site-level results, the data scientist at Consortium Hub merges these results into a single file. A generic and extensible format for site-level summary result is used to answer general epidemiology and clinical research questions (Figure 4). Then a data quality check is conducted. While use of a CDM in a large clinical data research network is a widely used approach to enable interoperable query development, a query formulated in 1 institution may not return accurate results in another due to variations in data integration and data quality differences. Several rounds of confirmations and checks with data analysts and clinical informaticians at each institution are often necessary to answer questions with confidence. There are many potential sources of errors, and Table 2 displays selected examples of data quality checks. The check types are based on the PEDSnet framework^17^ and revised to fit our project’s specific needs. The data scientist resolves issues together with the Lead Site and the Responding Site. When the aggregate results pass the quality control test, the Consortium Hub clinician conducts the final review to ensure its clinical relevancy. During several rounds of code releases and responses among the Lead Site and the Responding Site, database developers rewrite their ETL scripts to increase the accuracy of the query results. Finally, if the clinician approves the release of the result, the data scientist uploads the answer to the public website (https://covid19questions.org), notifies the requestor via an e-mail, and this completes the workflow. Quality improvement-related steps and data visualization are either semiautomated or manually conducted. ETL refresh, initial data quality check, and data aggregation are automated with scheduling scripts.

**Table 2.** Data quality checks and issues. Different data quality check types are enumerated together with real issues identified with this COVID-19 project

Check Type	Example of data quality issue
Date/time reversal	A condition/observation was recorded after discharge date
Extreme outlier	The hospital length of stay was greater than 80 days. The median length of stay ranged between 11 and 15 days in China and US studies
Gaps in data transformation	Discharge disposition and ICU departments were not transformed to OMOP
Loss of granularity during mapping	Invasive and noninvasive mechanical ventilation mapped to the same concept
Impossible events	Multiple death events occurred in different time points from multiple hospital encounters
Noncompliance to the output format	Header was missing in the predefined output .csv format, missing columns, shifted columns, and duplicate rows
Unexpected proportion	The percentage of current smokers was 65% at a certain site. The national percentage of smoking was 15.6% among male adults in 2018 US CDC data
Unexpected zero count	The number of patients who were taking any antihypertensives was zero
Unmatched group sum	The total sums of patient count in age groups and race groups were different even when all cell counts were greater than 10
Version mismatch	The version of the template query was revised after the query result was uploaded

### Federated regression

In addition to count queries, we also applied Grid Binary LOgistic REgression (GLORE)^15^ to compute the effect of the exposure variable on the outcome, adjusted for confounders, without sharing patient-level data, as this would increase the risk for a privacy breach. We rewrote the Newton-Raphson method to find the maximizer of the likelihood function of the parameters in logistic regression for horizontally partitioned datasets. Since the first and the second derivatives of the log likelihood functions are separable (ie, they can be partially calculated at each site), in each Newton-Raphson iteration, each client institute calculated a (*p *+* *1) dimensional vector of parameters, where *p* is the number of features in the model such as *age*, *sex*, and *race* and a (*p *+* *1) by (*p *+* *1) variance-covariance matrix; then JSON files containing these two objects are sent to the Consortium Hub. At each iteration, the Consortium Hub automatically updates the global coefficient vector and the variance-covariance matrix and sends them back to the clients.

## RESULTS

Between 12/11/2020 and 8/31/2020, our consortium had 928 255 tested patients for SARS-CoV-2, 59 074 diagnosed with COVID-19, with 19 022 hospitalized and 2591 deceased. Our public questions and answers portal (https://covid19questions.org) provides answers to research questions using several univariate or multivariate analyses, including potential associations between mortality and comorbidities; prehospitalization use of medications; laboratory values; and hospital events.

**Figure 5.**
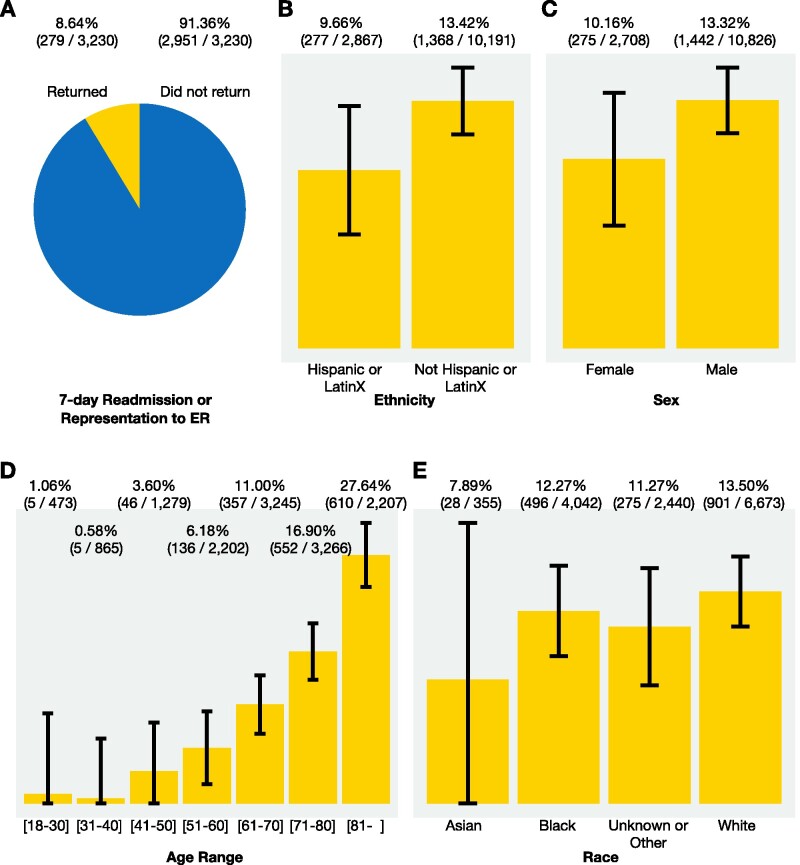
**Examples of 2 COVID-19 questions and answers: return to hospital and mortality.** (A) 8.6% of hospitalizations without an ICU admission resulted in the patient presenting to the emergency room or a hospital readmission within 7 days (data from 10 health systems). (B–E) Unadjusted mortality rates from aggregated results are shown with 95% confidence intervals (data from 10 health systems). Univariate analyses indicate that lower age, Hispanic ethnicity, and female sex (as recorded in the EHR) are associated with lower mortality for adult hospitalized COVID-19 patients.

**Figure 6.**
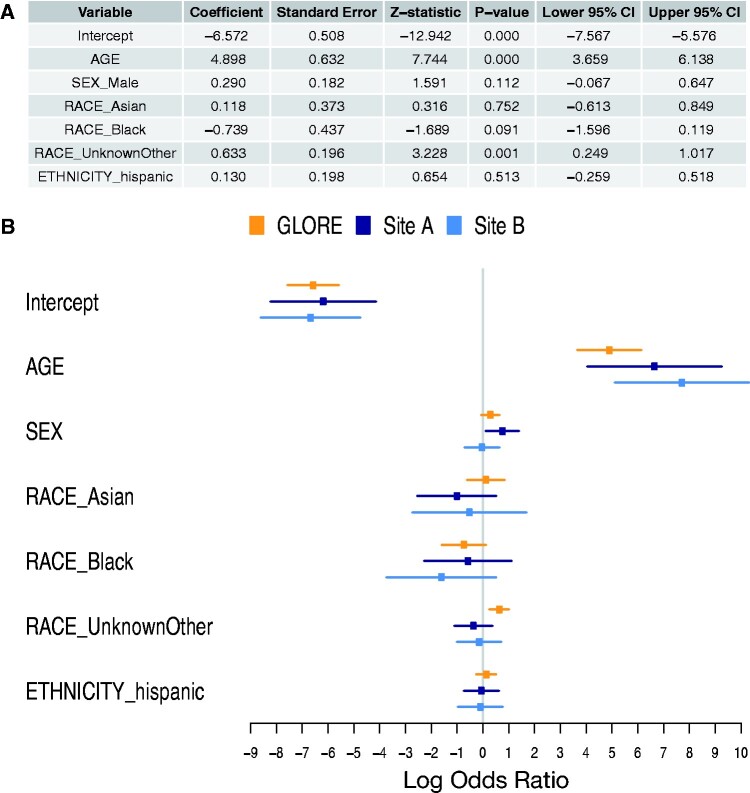
**Regression results.** (A) Adjusted effects from the Grid binary LOgistic REgression (GLORE) (15) federated logistic regression model (3146 patients from 8 health systems). The baselines were SEX=female, RACE=white, ETHNICITY=non-Hispanic. AGE (in years) was divided by 100. After adjustment via distributed logistic regression, AGE remains significant. (B) Results from local logistic regression performed at two sites are also shown for comparison with GLORE results.

For each question, we report on the number of participating institutions and the time period within which local queries were run. Figures 4–6 illustrate the answers.

Example 1. “Many adult COVID-19 patients who were hospitalized did not get admitted to the ICU and were discharged alive. How many returned to the hospital within a week, either to the emergency room or for another hospital stay?” This question is important both from the standpoint of understanding the natural course of disease and planning for needed resources. Although efforts are underway to understand postdischarge outcomes in COVID-19 infected patients, to date they have been limited to case series,^18^ modest sample sizes,^19^ or single-center or geographically concentrated health systems.^20^ These extant studies may also be hampered by fixed inclusion/exclusion criteria.^21^

Example 2. “Among adults hospitalized with COVID-19, how does the in-hospital mortality rate compare per subgroup (age, ethnicity, sex, and race)?” The answers from univariate analyses indicate that age, ethnicity, and sex are significant. A distributed logistic regression (Figure 6) shows, among these, that only age is significant. There is great interest and growing peer-reviewed literature on risk factors for COVID-19 mortality; the agility of our approach allows us to quickly rerun queries and rebuild models as new predictors become relevant and the understanding of the disease evolves.^20^^,^^22^^,^^23^

### Cohort and concept set

As questions frequently refer to the same subsets of patients, we developed electronic cohort definitions that facilitate our answers. We followed the US Centers for Disease Control and Prevention (CDC) guideline^24^ and the National COVID-19 Cohort Collaborative^25^ and Observational Health Data Sciences and Informatics (OHDSI) approaches^26^ to develop a cohort of hospitalization encounters for COVID-19 as a base for all inpatient questions. Through an iterative process among multiple sites, we developed a canonical SQL whose results match with that of the ground truth cohort definition. The intersection of the R2D2 canonical SQL, the private reference (ie, EHR- or registry-based) and the universal reference (ie, a positive polymerase chain reaction test for SARS-COV-2) was maximized for existing and new sites.

Figure 3A displays the electronic phenotyping of adults hospitalized with COVID-19 derived by the canonical SQL and stored procedure SQL scripts. Hospitalization encounters were identified by using the following concepts stored in the OMOP <VISIT_OCCURRENCE> table: Emergency Room and Inpatient Visit (Concept Id 262), Inpatient Visit (Concept Id 92021) or Intensive Care (Concept Id 32037). To enter the COVID-19 hospitalization cohort, all four inclusion criteria needed to be met:

1) a minimum age of 18 years at the date of hospitalization,

2–3) a hospitalization without a length of stay requirement on or after January 1, 2020, and

4) at least 1 occurrence of

- a positive viral test for SARS-CoV-2, or
- a COVID-19 related diagnosis between the interval of 21 days prior to hospitalization and hospital encounter discharge.

The following concepts of the OMOP <MEASUREMENT> table for the definition of a positive viral test for SARS-COV-2 were used:

1. the occurrence of the precoordinated measurement concept (Concept Name: 2019 novel coronavirus detected, Concept Id: 37310282), or
2. the occurrence of at least one concept for a SARS-CoV-2 viral test (eg, Concept Name: SARS-CoV-2 (COVID19) RNA [presence] in respiratory specimen by NAA with probe detection, Concept Id: 706163) and at least one *value_as_concept_id* for a positive result (eg, Concept Name: Positive, Concept Id: 45884084).

For identification of COVID-19 related diagnoses, we included the following ICD-10-CM Codes: Other coronavirus as the cause of diseases classified elsewhere (B97.29), COVID-19, virus identified (U07.1), Pneumonia (J12.89), Acute Bronchitis (J20.8), Lower Respiratory Infection (J22, J98.8), and Acute Respiratory Distress Syndrome (J80). Following 2 ICD-10-CM Official Coding and Reporting Guidelines released by CDC before and at/after April 1, 2020, we used diagnosis code aggregations to define a COVID-19 related diagnosis. An illness due to COVID-19 was specified if 1 of the ICD-10-CM codes (J12.89, J20.8, J22, J98.8, J80) was recorded in combination with either B97.29 (before April 1, 2020), or in combination with U07.1 (on/after April 1, 2020). These joint diagnosis codes needed to occur during the same hospitalization encounter, with a look back period of 21 days prior to hospitalization. We applied the same logic for mapped SNOMED concepts (261326, 260139, 4307774, 256451, 4195694, 320136, 4100065, 37311061). More ICD codes are detailed in Table 3. Precoordinated diagnoses codes (SNOMED, OMOP Extension) are shown in Supplementary Tables 1–3. Refinement of phenotypes was guided by chart review.

**Table 3.** Concept relationships between ICD10CM and SNOMED concepts. ICD10CM concepts and their mapped SNOMED concepts from the <CONDITION_OCCURRENCE> table. In OMOP CDM, ICD10CM concepts are non-standard concepts. Therefore, ICD10CM concepts are mapped to SNOMED-based standard concepts.^1^ These relationships are stored in the OMOP CDM <CONCEPT_RELATIONSHIP> table. In this case, each ICD10CM concept got the relationship_id = ‘Maps to,’ which directs to one SNOMED concept.

Concept Code 1 (ICD10CM)	Concept Name 1	Concept Id 1	Relationship Id	Concept Id 2	Concept Name 2	Concept Code 2 (SNOMED)
J12.89	Other viral pneumonia	45572161	‘Maps to’	261326	Viral pneumonia	75570004
J20.8	Acute bronchitis due to other specified organisms	35207965	‘Maps to’	260139	Acute bronchitis	10509002
J22	Unspecified acute lower respiratory infection	35207970	‘Maps to’	4307774	Acute lower respiratory tract infection	195742007
J40	Bronchitis, not specified as acute or chronic	35208013	‘Maps to’	256451	Bronchitis	32398004
J80	Acute respiratory distress syndrome	35208069	‘Maps to’	4195694	Acute respiratory distress syndrome	67782005
J98.8	Other specified respiratory disorders	35208108	‘Maps to’	320136	Disorder of respiratory system	50043002
B97.29	Other coronavirus as the cause of diseases classified elsewhere	45600471	‘Maps to’	4100065	Disease due to Coronaviridae	27619001
U07.1	Emergency use of U07.1 | Disease caused by severe acute respiratory syndrome coronavirus 2	702953	‘Maps to’	37311061	Disease caused by 2019-nCoV	840539006

Use cases of concept set are shown in Figure 3B. As the Responding Sites’ OMOP databases are not accessible to the Lead Site, a query developed at the Lead Site might miss a concept used in other sites. In such a case, the database analyst at the Responding Site notifies the Lead Site by creating a GitHub issue, with zero or unexpectedly low count or proportion in the results generated by the initial template query authored by the Lead Site. For example, in Figure 3B, during the Concept Set development for the quantitative laboratory measurement D-dimer, the responding site notified the Lead site about using another concept for D-dimer, (Concept ID: 3048540 instead of Concept ID: 3051714), returning values with a different measurement unit than the ones of the Lead Site (n/L instead of mg/L). Therefore, the Lead Site had to add the missing concept to the Concept Set and implemented logic to cover a measurement unit transformation. In the case of the Concept Set development for blood type, a responding site was missing concepts for blood type in its local OMOP CDM database. An ETL script was implemented to map EHR data to OMOP CDM. Sources of discrepancy were diverse; examples included unit differences in measurement values, differently mapped concepts, and noncompliance to the coding guideline. All SQL codes and concept sets for answered questions are publicly available from the GitHub repository: https://github.com/DBMI/R2D2-Public. The public repository is updated whenever a new question and its answer get posted on the public website. The similarities and the differences of our approach to other consortia are detailed in the Supplementary Material.

Supplementary Figure 1 shows the screen shot of the real example JSON file used during the GLORE run to answer the in-hospital mortality question. No patient-level information was shared or transferred between institutions. All clients repeatedly sent the updated JSON file to the Consortium Hub until the estimates stabilized or reached a predefined number of iterations. To enhance the security, the Consortium Hub server allowed (ie, “white listed”) only the preregistered IP addresses of client machines and opened the port only during the scheduled time window.

Several other questions and answers are shown in the portal. A novel governance structure (Figures 1 and 2) allows us to distribute the workload across various teams without relying on a traditional coordinating center, instead including a Consortium Hub. This approach keeps patient data in-house, simplifies data use agreements, avoids delegation of control of patient data to another institution, and allows any institution to benchmark its results to those produced by the consortium, since all questions and respective final, aggregated answers, database query code, concept definitions, and analytics code are made public. It complies with HIPAA,^27^ the Common Rule,^28^ the GDPR,^29^ and the California Consumer Privacy Act^30^ with regards to handling of patient data. Code sharing and public answers promote transparency and reproducibility without disclosing patient or institutional information.

## DISCUSSION

Our approach is practical and generalizable: The network can be repurposed to any other disease of interest, as it is not based exclusively on data elements deemed relevant for COVID-19. Because privacy protection is at the core of our network, a wide range of institutions can participate. We provide a rapidly deployable and reproducible alternative or complement to centralized registries of EHR data that allows healthcare institutions to stay in control of their data.

This study has advantages but also some limitations. The advantages are that we can, in relatively short time, publicly post answers, using data from a spectrum of institutions with different levels of information technology baselines and expertise in standardized data models and vocabularies, institutional policies, and state and federal regulations. Because we keep data locally and only consult data elements that are necessary to answer specific questions, this approach has a very low risk of privacy breach. However, for this reason, our approach does not provide answers in real time. We made this practical decision to quickly collect aggregate counts and statistics near real time within existing institutional policies and OMOP implementation to meet the clinical need of a rapidly spreading pandemic while preserving patient privacy. A real-time query with a fully automated process would be ideal, but this necessitates a long process of interinstitution agreement, amendments to the institutional policies, and a complete harmonization of EHR data across all sites. The use of OMOP CDM data is dependent on recurring ETL processes on each site, which presents a challenge to presenting real-time data. Additionally, as opposed to registries that typically focus on a single disease or condition, we have comparator data from other patients. Institutional privacy is also preserved because all public answers combine the aggregate data from at least three Responding Sites. Making concept definitions, query code, and results publicly available enhances reproducibility. A major advantage is that existing registries or consortia can serve as additional sites to help answer certain questions. However, the limitations are inherent from considering all sites equal when formulating a final answer, as regional or institutional practice variations are not represented in the answers. Additionally, the distributed nature of the consortium adds a requirement for educating some system leaders on distributed analytics. A specific limitation of our current consortium is the preponderance of institutions based in California: 67%, or 17.5% of COVID-19 patients. This was a convenience sample of organizations that had a history of collaboration. We are currently adding two new large health systems. One system is in the Northeast United States, and another is in the Southeast. To display changes over time and to help users compare our results to public results, new SQL code has been developed. Additionally, the increasing use of automated stored procedures will help reduce the manual process.

We believe that our Covid-19 Clinical Data Consult is a tool for achieving rapid and robust responses to COVID-19 questions submitted by the public or by researchers. We can achieve those goals by combining a transparent, privacy-preserving code-sharing workflow with the use of harmonized distributed data. A vision for the future in which there is convergence of data services would include interoperability with other efforts, including federated multivariate analyses across different consortiums (eg, R2D2, 4CE, and N3C).

## CONCLUSION

Instead of centralizing data at the Consortium Hub, we focus on interpreting and clarifying the research questions in order to determine the data elements required. Our teams analyze these data elements to generate aggregate statistics at the multiple institutions, documenting the specific version of SQL code executed at a specific time point to generate their answers. In addition to basic counts and proportions to adjust for confounders, we use distributed multivariate analyses to estimate risk-adjusted odds ratios. This is done in a synchronized fashion for iterative federated algorithms, such as one previously reported for building a logistic regression model. We have shown previously that a model obtained this way is identical to one built using data that are centralized in a single location. We made SQL codes, cohort definitions, and concept sets publicly available at https://github.com/DBMI/R2D2-Public. We invite other institutions, consortia, and registries worldwide to join us at https://covid19questions.org.

## FUNDING

This work was supported by the Gordon and Betty Moore Foundation #9639. The distributed analytics algorithm was funded by NIH-R01GM118609. Trainees were funded by NIH-T15LM011271. LN was funded by DIFUTURE (BMBF grant 01ZZ1804C).

## AUTHOR CONTRIBUTIONS

JK and LN contributed equally. JK had full access to all the data in the study. LOM contributed to the conception and design of the project. JK and LOM contributed to acquisition, analysis, and interpretation of data. JK, LN, and LOM drafted the manuscript. JK, LN, PP, MA, DSB, JND, LCH, XJ, KKK, MEM, DM, MJP, LS, SS, HX, KZ, and LOM provided the critical revision of the manuscript for important intellectual content along with administrative, technical, and material support. JK, MED, and LOM performed statistical analysis. LOM obtained funding.

## SUPPLEMENTARY MATERIAL

Supplementary material is available at *Journal of the American Medical Informatics Association* online.

## DATA AVAILABILITY STATEMENT

The aggregate data used in this article will be shared on reasonable request to the corresponding author.

## CONFLICT OF INTEREST STATEMENT

Hua Xu has financial interest at Melax Technologies Inc. Other authors declare no competing interests.

## Supplementary Material

ocab054_Supplementary_Data
