# Correction to: Privacy-protecting, reliable response data discovery using COVID-19 patient observations

**DOI:** 10.1093/jamia/ocad069

**Published:** 2023-04-18

This is a correction to: Jihoon Kim, Larissa Neumann, Paulina Paul, Michele E Day, Michael Aratow, Douglas S Bell, Jason N Doctor, Ludwig C Hinske, Xiaoqian Jiang, Katherine K Kim, Michael E Matheny, Daniella Meeker, Mark J Pletcher, Lisa M Schilling, Spencer SooHoo, Hua Xu, Kai Zheng, Lucila Ohno-Machado, for the R2D2 Consortium, Privacy-protecting, reliable response data discovery using COVID-19 patient observations, *Journal of the American Medical Informatics Association*, Volume 28, Issue 8, August 2021, Pages 1765–1776, https://doi.org/10.1093/jamia/ocab054

The originally published version of this manuscript inadvertently omitted listing the members of the R2D2 Consortium.

This error has been corrected.

