# Supplementary material for: Privacy-protecting, reliable response data discovery using COVID-19 patient observations
Source: J Am Med Inform Assoc. 2021 May 29;28(8):1765–76. doi: 10.1093/jamia/ocab054 (PMC8194878; doi:10.1093/jamia/ocab054)
Supplement: ocab054_Supplementary_Data [file ocab054_supplementary_data.docx]

**Supplementary Material**

Kim J, Neumann L, Paul P, et al. Privacy-Protecting, Reliable Response Data Discovery Using COVID-19 Patient Observations. JAMIA. 2021

**Supplementary Figure 1.** Transferred file in the federated logistic regression

**Supplementary Figure 2.** Extensible output format of site level results

**Supplementary Table 1.** Pre-coordinated diagnosis codes

**Supplementary Table 2.** Excluded diagnosis codes

**Supplementary Table 3.** Other excluded diagnosis codes

This supplementary material has been provided by the authors to give readers additional information about their work.

**Comparison to other consortia**

The R2D2 consortium is similar to N3C and OHDSI in that OMOP is used as a common data model. R2D2 and 4CE are similar in that both are distributed networks, do not disclose patient level data, and provide the time trend of COVID-19 related metrics on the public website. The R2D2 differs from other consortia (e.g., 4CE, N3C, and OHDSI) in five main points: (1) R2D2 allows the general public to ask questions, (2) Patient privacy is protected by sharing only aggregate level data and adoption of privacy-preserving federated regression method such as GLORE, and (3) our iterative workflow processes with a focus on decentralization and data quality checks lead to an amended data harmonization and high sensitivity and specificity in query results and increased site-level capacity building and independence, with support from the whole consortium. Similar to OHDSI, but unlike N3C and 4CE, (1) the use of EHR data empowers our network to answer questions which include both COVID-19 patient and non-COVID-19 or pre-COVID-19 patients (e.g., ‘For the previous 24 months, what are the monthly counts of encounters for breast cancer screening and are there disparities in the patterns as a result of the COVID-19 pandemic?’) and (2) full transparency is granted by sharing the finalized SQL codes on public webpages hosted through GitHub and their related results on our webpage. Additionally, the main difference with N3C is architectural: in R2D2 sites do not need to transmit data to a central repository. Similar to OHDSI and 4CE, we utilize a distributed approach in order to attend to the regulations at some institutions. While OHDSI is better suited to perform in-depth analyses for a certain number of questions over longer periods of time, research questions are developed inside the OHDSI consortia, R2D2 provides more shallow information by responding to a larger number of questions requested by the public.

Unlike other important initiatives such as ACT (used by several CTSAs) that also intend to respond to a larger number of questions in short time, the questions are expressed in natural language, making the approach more flexible although also more manual.

Despite these differences, multiple consortia could work together towards the common goal. First, sharing concept sets and SQL code through a public code repository would be one good starting place. Each consortium would simply download the script generated by another consortium and run it to reproduce and validate the early findings. Second, the documentation of data quality improvement as a knowledgebase would be another incentive for different consortia work together. In our experience, harmonization of measurement values like D-dimer and Vitamin-D took was a lengthy process of lab test review, running SQL codes in multiple versions, investigation to EHR system, updates to the ETL scripts, and brainstorming to understand different site-specific workflows. If each consortium could contribute to provide their best practice to the common knowledgebase, this would save time and efforts of other consortium and sites. For the same reason, in this rapidly generated study, we did not provide the validation results of our findings against those of other consortia. Instead, we shared the concept sets, SQL code, and aggregate results for others to validate their results on ours. Next step is working with other networks and make consorted efforts to develop codes and validate results together.

**Supplementary Figure 1. Transferred file in the federated logistic regression**. A real example of a transferred file in JSON format among the Consortium Hub and participating sites is shown to illustrate how patient level data are protected. The first row is a vector of standard deviations of the coefficients of the federated logistic regression. The second row is a vector of coefficients. The third row is a covariance matrix between features. At each iteration of the federated logistic regression, this JSON file is being transferred among sites until convergence or the process reaches the predetermined number of iterations. The values in JSON file above are used in the forest plot of Figure 2 in the main manuscript.

**Supplementary Figure 2. Extensible output format of site level results.** A 2-by-2 table of exposure-outcome association is implemented as 4-row format in SQL to store the binary exposure and the binary outcome question. The table will expand to add a covariate (Sex), a second outcome (Mechanical Ventilation), or another exposure (drinking status). A name-value format was adopted for clarity and efficiency during data quality check and aggregation.

**Supplementary Table 1. Pre-coordinated diagnosis codes.** Diagnosis codes (OMOP Extension, SNOMED) used to identify patients with COVID-19. At least one occurrence of the diagnosis codes during a hospital encounter with a look back period of 21 days prior to hospitalization captured the patient having a COVID-19 related diagnosis. In contrast to the joint diagnosis codes (ICD-10-CM, SNOMED) mentioned in **Figure 3A**, there was no further applied logic.

| **Concept Class Id** | **Vocabulary Id** | **Concept Code** | **Concept Id** | **Concept Name** |
| --- | --- | --- | --- | --- |
| Clinical Finding | OMOP Extension | OMOP4873906 | 756023 | Acute bronchitis due to COVID-19 |
| Clinical Finding | OMOP Extension | OMOP4873911 | 756044 | Acute respiratory distress syndrome (ARDS) due to COVID-19 |
| Clinical Finding | OMOP Extension | OMOP4873910 | 756061 | Asymptomatic COVID-19 |
| Clinical Finding | OMOP Extension | OMOP4873909 | 756031 | Bronchitis due to COVID-19 |
| Clinical Finding | SNOMED | 1240561000000108 | 37310284 | Encephalopathy caused by 2019 novel coronavirus |
| Clinical Finding | SNOMED | 1240571000000101 | 37310283 | Gastroenteritis caused by 2019 novel coronavirus |
| Clinical Finding | OMOP Extension | OMOP4873908 | 756081 | Infection of lower respiratory tract due to COVID-19 |
| Clinical Finding | SNOMED | 1240541000000107 | 37310286 | Infection of upper respiratory tract caused by 2019 novel coronavirus |
| Clinical Finding | SNOMED | 1240531000000103 | 37310287 | Myocarditis caused by 2019 novel coronavirus |
| Clinical Finding | SNOMED | 1240521000000100 | 37310254 | Otitis media caused by 2019 novel coronavirus |
| Clinical Finding | SNOMED | 1240551000000105 | 37310285 | Pneumonia caused by 2019 novel coronavirus |
| Clinical Finding | OMOP Extension | OMOP4873907 | 756039 | Respiratory infection due to COVID-19 |

**Supplementary Table 2. Excluded diagnosis codes.** These ICD-10-CM Codes, mentioned in CDC guideline, were excluded given the high count of false positive COVID-19 patients.

| **ICD-10-CM Code** | **Description** |
| --- | --- |
| R05 | Cough |
| R06.02 | Shortness of breath |
| R50.9 | Fever, unspecified |
| Z20.828 | Contact with and (suspected) exposure to other viral communicable diseases |

**Supplementary Table 3. Other excluded diagnosis codes.** These ICD10CM and SNOMED Concepts were mentioned in N3C - COVID-19 Phenotype Documentation, Version 1.6 (Last updated 6/5/2020) but were excluded from our study, as the count of false positives COVID-19 patients was too high.

| **Concept Class Id** | **Vocabulary Id** | **Concept Code** | **Concept Id** | **Concept Name** |
| --- | --- | --- | --- | --- |
| Condition | ICD10CM | J96.2 | 35208101 | Acute and chronic respiratory failure |
| Condition | ICD10CM | J96.22 | 45581868 | Acute and chronic respiratory failure with hypercapnia |
| Condition | ICD10CM | J96.21 | 45543283 | Acute and chronic respiratory failure with hypoxia |
| Condition | ICD10CM | J96.20 | 45596290 | Acute and chronic respiratory failure, unspecified whether with hypoxia or hypercapnia |
| Condition | ICD10CM | J21 | 1569471 | Acute bronchiolitis |
| Condition | ICD10CM | J21.1 | 920135 | Acute bronchiolitis due to human metapneumovirus |
| Condition | ICD10CM | J21.8 | 35207968 | Acute bronchiolitis due to other specified organisms |
| Condition | ICD10CM | J21.0 | 35207967 | Acute bronchiolitis due to respiratory syncytial virus |
| Condition | ICD10CM | J21.9 | 35207969 | Acute bronchiolitis, unspecified |
| Condition | ICD10CM | J20 | 1569470 | Acute bronchitis |
| Condition | ICD10CM | J20.3 | 35207960 | Acute bronchitis due to coxsackievirus |
| Condition | ICD10CM | J20.7 | 35207964 | Acute bronchitis due to echovirus |
| Condition | ICD10CM | J20.1 | 35207958 | Acute bronchitis due to Hemophilus influenzae |
| Condition | ICD10CM | J20.0 | 35207957 | Acute bronchitis due to Mycoplasma pneumoniae |
| Condition | ICD10CM | J20.8 | 35207965 | Acute bronchitis due to other specified organisms |
| Condition | ICD10CM | J20.4 | 35207961 | Acute bronchitis due to parainfluenza virus |
| Condition | ICD10CM | J20.5 | 35207962 | Acute bronchitis due to respiratory syncytial virus |
| Condition | ICD10CM | J20.6 | 35207963 | Acute bronchitis due to rhinovirus |
| Condition | ICD10CM | J20.2 | 35207959 | Acute bronchitis due to streptococcus |
| Condition | ICD10CM | J20.9 | 35207966 | Acute bronchitis, unspecified |
| Condition | ICD10CM | R06.03 | 1326788 | Acute respiratory distress |
| Condition | ICD10CM | J80 | 35208069 | Acute respiratory distress syndrome |
| Condition | ICD10CM | J96.0 | 35208099 | Acute respiratory failure |
| Condition | ICD10CM | J96.02 | 45596289 | Acute respiratory failure with hypercapnia |
| Condition | ICD10CM | J96.01 | 45567283 | Acute respiratory failure with hypoxia |
| Condition | ICD10CM | J96.00 | 45605906 | Acute respiratory failure, unspecified whether with hypoxia or hypercapnia |
| Condition | ICD10CM | J06.9 | 35207929 | Acute upper respiratory infection, unspecified |
| Condition | ICD10CM | J12.0 | 35207932 | Adenoviral pneumonia |
| Condition | ICD10CM | R43.0 | 35211351 | Anosmia |
| Condition | ICD10CM | J40 | 35208013 | Bronchitis, not specified as acute or chronic |
| Condition | ICD10CM | J18.0 | 35207952 | Bronchopneumonia, unspecified organism |
| **Concept Class Id** | **Vocabulary Id** | **Concept Code** | **Concept Id** | **Concept Name** |
| Condition | ICD10CM | R07.1 | 35211284 | Chest pain on breathing |
| Condition | ICD10CM | R68.83 | 45577807 | Chills (without fever) |
| Condition | ICD10CM | J96.1 | 35208100 | Chronic respiratory failure |
| Condition | ICD10CM | J96.12 | 45572177 | Chronic respiratory failure with hypercapnia |
| Condition | ICD10CM | J96.11 | 45538489 | Chronic respiratory failure with hypoxia |
| Condition | ICD10CM | J96.10 | 45548131 | Chronic respiratory failure, unspecified whether with hypoxia or hypercapnia |
| Observation | ICD10CM | Z20.828 | 45542411 | Contact with and (suspected) exposure to other viral communicable diseases |
| Condition | ICD10CM | B34.2 | 35205800 | Coronavirus infection, unspecified |
| Condition | ICD10CM | R05 | 35211275 | Cough |
| Condition | ICD10CM | R50.2 | 35211385 | Drug induced fever |
| Condition | ICD10CM | R06.0 | 1572191 | Dyspnea |
| Condition | ICD10CM | R06.00 | 45587496 | Dyspnea, unspecified |
| Condition | ICD10CM | R50.84 | 45597190 | Febrile nonhemolytic transfusion reaction |
| Condition | ICD10CM | R50 | 1572254 | Fever of other and unknown origin |
| Condition | ICD10CM | R50.81 | 45606818 | Fever presenting with conditions classified elsewhere |
| Condition | ICD10CM | R50.9 | 35211387 | Fever, unspecified |
| Condition | ICD10CM | J12.3 | 35207935 | Human metapneumovirus pneumonia |
| Condition | ICD10CM | J18.2 | 35207954 | Hypostatic pneumonia, unspecified organism |
| Condition | ICD10CM | J18.1 | 35207953 | Lobar pneumonia, unspecified organism |
| Condition | ICD10CM | R06.01 | 45597165 | Orthopnea |
| Condition | ICD10CM | R06.09 | 45548944 | Other forms of dyspnea |
| Condition | ICD10CM | J18.8 | 35207955 | Other pneumonia, unspecified organism |
| Condition | ICD10CM | R50.8 | 35211386 | Other specified fever |
| Condition | ICD10CM | J98.8 | 35208108 | Other specified respiratory disorders |
| Condition | ICD10CM | J12.8 | 35207936 | Other viral pneumonia |
| Condition | ICD10CM | R43.2 | 35211353 | Parageusia |
| Condition | ICD10CM | J12.2 | 35207934 | Parainfluenza virus pneumonia |
| Condition | ICD10CM | J12.81 | 45567260 | Pneumonia due to SARS-associated coronavirus |
| Condition | ICD10CM | J18.9 | 35207956 | Pneumonia, unspecified organism |
| Condition | ICD10CM | J18 | 1569469 | Pneumonia, unspecified organism |
| Condition | ICD10CM | R50.82 | 45597189 | Postprocedural fever |
| Condition | ICD10CM | R50.83 | 45592424 | Postvaccination fever |
| Condition | ICD10CM | J96 | 1569515 | Respiratory failure, not elsewhere classified |
| Condition | ICD10CM | J96.9 | 35208102 | Respiratory failure, unspecified |
| Condition | ICD10CM | J96.92 | 45533563 | Respiratory failure, unspecified with hypercapnia |
| Condition | ICD10CM | J96.91 | 45605907 | Respiratory failure, unspecified with hypoxia |
| **Concept Class Id** | **Vocabulary Id** | **Concept Code** | **Concept Id** | **Concept Name** |
| Condition | ICD10CM | J96.90 | 45567284 | Respiratory failure, unspecified, unspecified whether with hypoxia or hypercapnia |
| Condition | ICD10CM | J12.1 | 35207933 | Respiratory syncytial virus pneumonia |
| Condition | ICD10CM | R06.02 | 45534422 | Shortness of breath |
| Condition | ICD10CM | J12 | 1569465 | Viral pneumonia, not elsewhere classified |
| Condition | ICD10CM | J12.9 | 35207937 | Viral pneumonia, unspecified |
| Condition | SNOMED | 75483001 | 442555 | Breathing painful |
| Condition | SNOMED | 161940008 | 4059022 | Breathless - mild exertion |
| Condition | SNOMED | 161939006 | 4059021 | Breathless - moderate exertion |
| Condition | SNOMED | 161855003 | 4059003 | C/O shivering |
| Condition | SNOMED | 274664007 | 4168213 | Chest pain on breathing |
| Condition | SNOMED | 43724002 | 434490 | Chill |
| Condition | SNOMED | 49727002 | 254761 | Cough |
| Condition | SNOMED | 135883003 | 4048098 | Cough with fever |
| Condition | SNOMED | 11833005 | 4038519 | Dry cough |
| Condition | SNOMED | 267036007 | 312437 | Dyspnea |
| Condition | SNOMED | 161941007 | 4060052 | Dyspnea at rest |
| Condition | SNOMED | 60845006 | 4263848 | Dyspnea on exertion |
| Observation | SNOMED | 840546002 | 37311059 | Exposure to 2019 novel coronavirus |
| Condition | SNOMED | 103001002 | 4011766 | Feeling feverish |
| Condition | SNOMED | 386661006 | 437663 | Fever |
| Measurement | SNOMED | 426000000 | 4141062 | Fever greater than 100.4 Fahrenheit |
| Condition | SNOMED | 274640006 | 4164645 | Fever with chills |
| Condition | SNOMED | 23141003 | 4047610 | Gasping for breath |
| Condition | SNOMED | 409702008 | 4260205 | Hyperpyrexia |
| Condition | SNOMED | 44169009 | 4185711 | Loss of sense of smell |
| Condition | SNOMED | 36955009 | 4289517 | Loss of taste |
| Condition | SNOMED | 426976009 | 4140453 | Pain provoked by breathing |
| Condition | SNOMED | 247410004 | 4090569 | Painful cough |
| Condition | SNOMED | 284523002 | 4109381 | Persistent cough |
| Condition | SNOMED | 2237002 | 4330445 | Pleuritic pain |
| Condition | SNOMED | 28743005 | 4102774 | Productive cough |
